# Paragraph-level attention based deep model for chapter segmentation

**DOI:** 10.7717/peerj-cs.1003

**Published:** 2022-06-10

**Authors:** Paveen Virameteekul

**Affiliations:** Department of Computer Science & Engineering, Shanghai Jiao Tong University, Minhang, Shanghai, China

**Keywords:** Machine learning, Neural networks, Text segmentation, XLNet, Supervised learning, Convolutional neural network, BERT, Natural language processing

## Abstract

Books are usually divided into chapters and sections. Correctly and automatically recognizing chapter boundaries can work as a proxy when segmenting long texts (a more general task). Book chapters can be easily segmented by humans, but automatic segregation is more challenging because the data is semi-structured. Since the concept of language is prone to ambiguity, it is essential to identify the relationship between the words in each paragraph and classify each consecutive paragraph based on their respective relationships with one another. Although researchers have designed deep learning-based models to solve this problem, these approaches have not considered the paragraph-level semantics among the consecutive paragraphs. In this article, we propose a novel deep learning-based method to segment book chapters that uses paragraph-level semantics and an attention mechanism. We first utilized a pre-trained XLNet model connected to a convolutional neural network (CNN) to extract the semantic meaning of each paragraph. Then, we measured the similarities in the semantics of each paragraph and designed an attention mechanism to inject the similarity information in order to better predict the chapter boundaries. The experimental results indicated that the performance of our proposed method can surpass those of other state-of-the-art (SOTA) methods for chapter segmentation on public datasets (the proposed model achieved an F1 score of 0.8856, outperforming the Bidirectional Encoder Representations from Transformers (BERT) model’s F1 score of 0.6640). The ablation study also illustrated that the paragraph-level attention mechanism could produce a significant increase in performance.

## Introduction

Nowadays, there is a huge amount of books and most of them are unstructured data. Loading or rendering the massive data at once can make the applications slow. One technique to solve this problem is to segment those data into multiple parts, and use only the necessities. As a consequence, the applications will not be easily overloaded. Therefore, our goal is to convert unstructured data into semi-structured data to avoid the previously mentioned problems. One commonly seen application is a reading application, which contains numerous books. Detection and segmentation of chapter boundaries can reduce the reading application cost.

It is quite important to automatically understand and analyze books. One important task in understanding and analyzing a book is to correctly segment its chapters. Typically, books are divided using unique keywords: chapters, parts, and/or Roman numerals. However, these keywords may be lost due to data corruption and thus are not suitable when used to determine chapter boundaries. Different approaches have been proposed to solve this problem.

Text segmentation (Pethe, Kim & Skiena, 2020; Haruechaiyasak, Kongyoung & Dailey, 2008; Koshorek et al., 2018; Li et al., 2020; Lukasik et al., 2020; Nguyen et al., 2021) is a method that is typically used to segment chapters by separating text into multiple segments or boundaries. It has also been used in many natural language processing tasks, such as word tokenization, text summarization (Hulliyah & Kusuma, 2010; Awasthi et al., 2021), question answering prediction (Wang, Ling & Hu, 2019), and machine translation (Kong, Zhang & Hovy, 2020; Gupta et al., 2021; Budiwati & Aritsugi, 2022).

Recent research has proposed the building of a deep-learning system to automatically identify chapter boundaries. For example, Pethe, Kim & Skiena (2020) proposed a Chapter Captor used to correctly recognize chapter breakpoints in novels. They proposed using Bidirectional Encoder Representations from Transformers (Devlin et al., 2018) to learn the semantic features and generate token-wise softmax probabilities. BERT is an auto-encoder language model that reconstructs original data from any corrupted inputs. Its advantage is that it can learn the context from both forward and backward directions. However, such auto-encoder language model algorithms cause the masked tokens in the pre-training to become non-existent during the fine-tuning stages. This ultimately leads to a pre-training and fine-tuning discrepancy (Yang et al., 2019). Another disadvantage is that each unmasked token is independent to the masked tokens, which means that the relationship between the masked tokens is ignored by the auto-encoder language model’s algorithms.

Considering these limitations of BERT (Pethe, Kim & Skiena, 2020), we instead propose the use of the XLNet model (Yang et al., 2019). The XLNet model is a generalized auto-regressive model that uses a permutation language model that helps the model learn a bidirectional context. Unlike BERT which tries to reconstruct the original data from the corrupted input, XLNet does not rely on data corruption. Due to the use of permutation, XLNet models learn to predict from all positions on both sides. Because of these advantages, it has been hypothesized that XLNet can overcome the BERT method in predicting the output for chapter segmentation (Yang et al., 2019).

Previous researchers found that information is mostly processed by token-level networks that cannot adequately reflect the basic unit of books, *i.e*., paragraphs. This is particularly apparent for the task of chapter segmentation because we have to learn the described topic differences across two chapters, which requires paragraph-level understanding. As a result, we propose using an attention mechanism to aggregate context information at the paragraph level.

This article proposes a novel deep learning-based method to segment book chapters. Our algorithm focused on extracting a paragraph-level attention module by utilizing a pre-trained XLNet model together with the convolutional neural network (CNN) and an attention module for paragraph-wise context information. The XLNet model performed word embedding, and the CNN simultaneously extracted features of the inputs. We then got semantic meaning from each paragraph following the self-attention layer. We calculated the distance between semantics from each paragraph and assumed that the closer the semantics, the more likely that the two paragraphs were in the same chapter, and *vice versa*. Then, we improved the model by the ensemble model using different positive and negative ratio labels of 0 and 1. The paragraph-level attention model had an F1 score of 0.8084, while the ensemble method improved its F1 score to 0.8856. Our results were then compared with the best methods found in Pethe, Kim & Skiena (2020) (BERT Break Point Prediction Model) and the F1 confusion matrix, as Their’s methods were considered to be the best practice. Pethe, Kim & Skiena (2020) used a pre-trained BERT model for the Next Sentence Prediction task combining with the dynamic programming algorithm. The BERT Break Point Prediction model (Pethe, Kim & Skiena, 2020) successfully competed with all baseline models, including the C99 algorithm from Choi (2000), the three-layer baseline perceptron model with 300 neurons in each layer (Badjatiya et al., 2018) and trained word2vec embeddings from Mikolov et al. (2013), and the neural model described by Badjatiya et al. (2018) that used long short-term memory (LSTM). Ultimately, we used the BERT model in this article as the baseline model for comparative analysis.

## Related Work

### Document segmentation

Book and document segmentation have been substantially explored and can be categorized into two mainstreams: computer vision-based methods and natural language processing-based methods.

One computer vision method, DocParser (Rausch et al., 2019), provides an end-to-end system that parses documents into a hierarchical structure. A CNN is used to segment documents into boundary boxes, which include paragraphs, table cells, and figures, by processing the document images as inputs. Wang, Li & Fang (2020) used few-shot learning to extract an image’s features with the advantage of using less data. Using optical character recognition (OCR) to extract books is another method shown in the ICDAR 2013 competition on Book Structure Extraction (Doucet et al., 2013).

On the other hand, natural language processing can segment books and documents using only text information. Koshorek et al. (2018) text segmentation model provided a bidirectional LSTM and sentence embedding model to extract and label text from the Wiki-727 dataset. McConnaughey, Dai & Bamman (2017) compared three different models used to automatically label book structures, and found that bidirectional LSTM was the best model. Raghavan, Kovashka & Mooney (2010) explored how to identify the author of a document using probabilistic context-free grammar. Name entity recognition (NER) (Li, Shang & Chen, 2021a) is used to recognize the type of the text on a passage, such as a person, location, and time. Next, neural name entity boundary detection (Li, Sun & Ma, 2021b) is another algorithm to detect the start and end boundaries of an entity without knowing the entity types by using BdryBot, a recurrent neural network encoder-decoder framework. Although all of the above techniques can be applied to chapter segmentation tasks, this article will propose novel methods.

### BERT

BERT is an auto-encoder language model that uses bidirectional pre-training and masked language models. It restores the initial data from any corrupted inputs and can learn context in bi-directions. However, BERT’s algorithm hides the masked tokens during pre-training. This creates a disparity between the pre-training and fine-tuning stages (Yang et al., 2019; Gao et al., 2019; Ye et al., 2021). Another disadvantage is that each unmasked token is independent of the masked tokens, which means the relationship between the masked tokens are ignored by the auto-encoder language model’s algorithms.

### XLNet

The XLNet model differs from the previous techniques because it is not only a transferring learning method, but it is also an auto-regressive pre-training model that enables the model to learn bidirectional contexts using a permutation order. The XLNet also utilizes an idea of relative position encoding and segment recurrence mechanism, unlike BERT which uses the absolute position embedding. With these two techniques, the model can compute the query stream without knowing the factorization order from the previous segments. Because of this advantage, the XLNet method has proven that this method outperforms the BERT model on 20 NLP tasks and achieves state-of-the-art results (Yang et al., 2019; Wang et al., 2021; Gong, Jin & Zhang, 2019). Therefore, the XLnet is chosen as a backbone.

### CNN

A CNN is a method used to learn data characteristics (Wang & Gang, 2018; Albawi, Mohammed & Al-Zawi, 2017). It consists of multiple layers of the neural network method. CNNs have been frequently used for image recognition and classification (Chauhan, Ghanshala & Joshi, 2018; Krizhevsky, Sutskever & Hinton, 2017) as well as text classification (Song, Geng & Li, 2019).

### Attention mechanism

Self-attention (Vaswani et al., 2017) is an attention mechanism that takes n inputs and returns n outputs. The inputs interact with each other in different positions and determine the relationship between each input. The outputs are then aggregated from the interaction and attention scores. Self-attention has been used in many tasks such as dependency parsing (Martins & Kreutzer, 2017), emoji prediction (Barbieri et al., 2018), and machine translation (Bahdanau, Cho & Bengio, 2015; Clark et al., 2019).

Pethe, Kim & Skiena (2020) provided a combination of rule-based and neural inferences in order to create their own label dataset from Project Gutenberg. They utilized a pre-trained BERT Next Sentence Prediction model and dynamic programming algorithm to segment books into chapters that were then used as a baseline for comparative analysis in this article.

## Dataset

This article used the dataset from Pethe, Kim & Skiena (2020), which contained 9,141 labeled books. This dataset extracted all features from the html file provided by the Project Gutenberg database into Gutenberg header, front matter, body, and Gutenberg footer categories.

This article focuses solely on the process of chapter segmentation from the datasets of multiple books. Therefore, other unused elements were not included and have been eliminated, and only the body parts that contained paragraphs remain. The remaining relevant data were then allocated into proportions and split into training data and testing data with a ratio of 60:40 (60 being the training data and 40 being the testing data). The training data were then further split into subsets of training data and validating data with a ratio of 90:10.

## Methods

**Figure 1:**
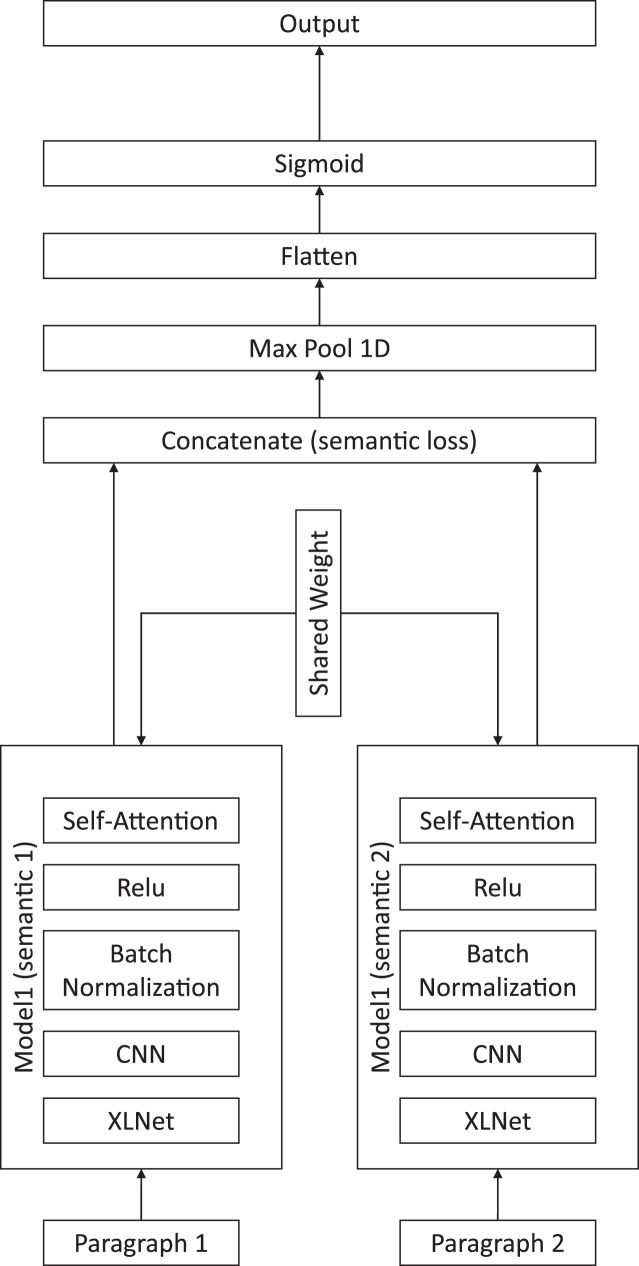
Illustration of the proposed chapter segmentation method.

Our proposed method is presented in Fig. 1, and includes a XLNet for embedding sentences, a CNN for feature extraction, an attention module for paragraph-level semantic aggregation, and the final classification module.

Refer to (1), given that *P* is a sequence of paragraphs, where *p*_*i*_ refers to the paragraph number and *n* refers to the total number of paragraphs. Refer to (2), let *X* denote the input, which will be a list of a pair of two consecutive paragraphs. Let *Y* be a sequence that labels between all consecutive paragraphs whether those two consecutive paragraphs are in the same chapter or not, where *y*_*i*_ is the label between paragraphs *p*_*i*_ and *p*_*i*+1_ as shown in (3). If two consecutive paragraphs are in the same chapter, the label is 0. If two consecutive paragraphs are not in the same chapter, the label is 1.

(1) $$P = {p_0},\ldots,{p_n}$$

(2) $$X = [({p_0},{p_1}),({p_1},{p_2}),\ldots,({p_{n - 1}},{p_n})]$$

(3) $$Y = {y_0},\ldots,{y_n}$$

### XLNet and CNN for feature learning

XLNet uses a combination of an autoregressive (AR) language model and autoencoding (AE). Yang et al. (2019) states that XLNet uses “all possible permutations of the factorization order”, which means it can avoid the limitations of AR models that only train on unidirectional contexts. Furthermore, XLNet does not rely on data corruption, unlike AE base training, which aims to predict only from corrupted data.

According to Yang et al. (2019)’s theory, the input format for the pre-trained XLNet is (4).

(4) $${Input} = {[A,SEP,CLS]}$$

where “*SEP*” and “*CLS*” are special symbols and “*A*,” is a segment. In this case, “*A*” is the paragraph for which we want to determine the semantic meaning.

The words in each paragraph are tokensized using an XLNet pretrain tokenizer. The inputs are equal to two consecutive paragraphs, and each paragraph may be different in length. To avoid an incompatible paragraph size, the boundary of the maximum length of each input paragraph was set at 254 words.

For example, if paragraph A contains fewer than the maximum words of 254 words, then paragraph A will be concatenated with the consecutive paragraphs that are set prior to A until the number of words reaches the maximum. However, if the words in paragraph A are beyond the maximum set-limit, then the algorithm will remove the words at the first index until there are only 254 words left.

Similar to paragraph A in each input, if the number of words in paragraph B in each input is fewer than the maximum length, words will be added to the paragraph after paragraph B until the paragraph reaches the maximum number of words. If the number of words in paragraph B is greater than the maximum set-limit, the algorithm will remove the words at the last index until there are only 254 words left.

We used the CNN to extract the text features from the XLNet’s matrix output. The average number of words in an English sentence is 15–20 words. Therefore, the kernel size was set to seven, which is half of the average sentence length. The convolution kernel was slid two words at a time. Finally, we filtered the dimension of the output space to 64. After the concatenation of the CNN and attention layer, we used max pooling to extract each concatenate CNN filter.

### Paragraph level attention for chapter understanding

The semantic features learned by XLNet and CNN were actually mainly at the token level. However, paragraph-level information is important for better understanding topics in the chapters. Thus, we proposed a paragraph-level attention module.

First, we created a paragraph-level attention module by connecting the XLNet pretrained model, CNN, batch normalization, activation, and self-attention layers together. The purpose of this module was to determine the semantic meaning of the input paragraphs. Once we determined the matrix of the semantic meaning of the paragraphs, the model concatenated the semantic meanings of the two consecutive paragraphs and calculated the contrastive loss, as shown in (5).

(5) $$L({S_1},{S_2}) = max(0,||{S_1} - {S_2}{||^2})$$

where *L* is the loss or the distance between the semantics of the two paragraphs. *S*_1_ and *S*_2_ refer to the semantic meanings of the two consecutive paragraphs.

After calculating the semantic loss of the two paragraphs, we assumed that a small loss indicated that the two paragraphs were in the same chapter since they had similar meanings. Therefore, a large semantic loss suggested that two paragraphs were not in the same chapter. After concatenating the two paragraphs’ semantic meanings, the model created a down sample of the feature map using the max pool layer. We then flattened the result into the sigmoid activation layer to classify the labels between the two consecutive input paragraphs. The flow of the model is shown in Fig. 1.

The training data were balanced with label 1, which designated a pair of consecutive paragraphs that were not in the same chapter, while label 0 was used for a pair of paragraphs that were in the same chapter. There was a ratio of 1:2, with label 0 being double that of label 1. The reason for this ratio was that book chapters usually do not only consist of a single paragraph.

The model was evaluated by looking at all pairs of consecutive paragraphs from books and counting all correct predictions, including label 0 and label 1. The paragraph-level attention model gave an accuracy score of 0.9920, precision score of 0.6900, recall score of 0.9759, and F1 score of 0.8084.

### Ensemble paragraph level attention

**Figure 2:**
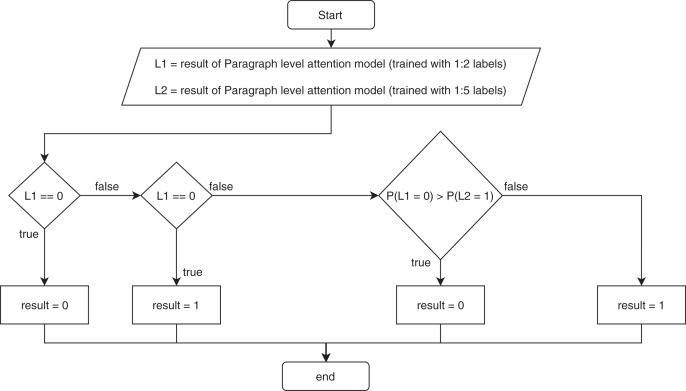
Flowchart of the ensemble for the proposed model.

The paragraph level attention model showed low precision compared to the recall, indicating that the model made many false positive predictions. Therefore, we improved the precision by making an ensemble model that kept the recall at about the same level. First, we added more training data. Next, we increased the ratio of the different chapter labels to the same chapter labels from 1 to 2 to a ratio of 1 to 5. Then, we trained the model using the new training set. Finally, we combined the previous paragraph-level attention model with the new training paragraph-level attention model using a rule: if the previous model predicted 0, meaning two paragraphs are in the same chapter, the result was 0; however, if the previous model predicted 1, meaning two paragraphs are in different chapters, then we used the new trained model to predict the result. If the new trained model predicted the label 1, then the result was 1. If the new trained model predicted 0, then we compared the probability of it being 0 from the previous model to the probability of it being 1 from the new trained paragraph-level attention model. The result was chosen from the model with the higher probability. Figure 2 illustrates the ensemble paragraph-level model.

## Experiments and Results

### Evaluation metrics

Matrices were generated for each model where they were compared using accuracy, precision, recall, and F1 scores as the measuring criteria. The higher the results of these measuring criteria, the better the performance of the models. The F1 score categorized the models with label 1 as positive, and 0 as negative.

### Precision

We divided the number of true positive predictions from the model by the total number of positive scores (Dalianis, 2018; Awan et al., 2020).

### Recall

We divided the number of true positive predictions from the model by the total number of actual positive scores (Dalianis, 2018; Awan et al., 2020).

### F1 score

The F1 score is a harmonic mean between the precision and recall scores (Dalianis, 2018; Awan et al., 2020). Therefore, it is a way to evaluate the balance of both precision and recall. The F1 score is a better form of measurement for the incorrectly classified class than the accuracy score. The F1 equation is shown in (6).

(6) $${\rm F1} = \displaystyle{{2*precision*recall} \over {precision + recall}}$$

### Compared with state-of-the-art methods

The models were tested in a real situation where the number of output labels were not balanced in order to demonstrate their true performance. Table 1 shows the accuracy, precision, recall, and F1 scores for the proposed model.

**Table 1:** Evaluation of the accuracy, precision, recall, and F1 scores for the proposed model.

Model	Accuracy	Precision	Recall	F1
Naive Bayes	0.1208	0.2784	0.0055	0.0108
SVM	0.0854	0.0106	0.5636	0.0209
BERT Break Point Prediction (Pethe, Kim & Skiena, 2020)	0.7719	0.6661	0.6618	0.6640
Paragraph level attention model (1:2)	0.9920	0.6900	0.9759	0.8084
Paragraph level attention model (1:5)	0.9953	0.8043	0.9637	0.8768
Ensemble paragraph level attention model	0.9957	0.8177	0.9659	0.8856

**Note:**

The paragraph-level attention model (1:2) is for the ratio of one to two training data labels. The paragraph-level attention model (1:5) is for the ratio of one to five training data labels. The ensemble paragraph-level attention model displayed the best result.

The paragraph-level attention model achieved an accuracy score of 0.9920. This model also reached a precision score of 0.6900, recall score of 0.9759, and F1 score of 0.8084. It should be noted that this particular model achieved higher precision, recall, and F1 scores than the BERT Break Point Prediction model.

Before we ensembled the paragraph-level attention model, we trained the paragraph-level attention model with another ratio of labels. The ratio of the two paragraphs in different chapters to the two paragraphs in the same chapter was 1 to 5. This new trained model had an F1 score of 0.8768, which was a 7% improvement from the model trained with a ratio of 1 to 2 labels. The new trained paragraph-level attention model also had improved accuracy (0.9953) and precision (0.8043) scores, but the recall score decreased by 1% to 0.9637.

On the other hand, the ensemble paragraph-level attention model’s results improved when compared to the paragraph-level attention model across both ratios. The ensemble paragraph-level attention model had an accuracy of 0.9957. Out of all the models, the ensemble paragraph-level attention model achieved the highest level of precision at 0.8177, with the second highest in recall at 0.9659. The ensemble paragraph-level attention model received the highest F1 score of 0.8856, which was more than a 7% improvement compared to the paragraph-level attention model and an approximately 20% improvement when compared to the baseline model.

Traditional machine learning models, such as Naive Bayes and Support-vector machine (SVM), are commonly used for classification tasks. A Naive Bayes (Webb, 2010) uses a probabilistic mechanism to estimate the probability of each class *y*, which considers a chapter break-point or not, given by *X* features. Multinomial Naive Bayes is used for comparing with the purposed method. By concatenating two consecutive paragraphs as an input, the model returns the probability of 0 and 1. The result shows a low overall score. The Naive Bayes model receives an accuracy of 0.1208, precision of 0.2784, recall of 0.0055, and F1 score of 0.0108. The Naive Bayes model runs fast and is suitable for a classification task; however, it assumes that all features are independent. which does not fit with the book’s contents. In consequence, it causes a low accuracy. Next, the SVM is an algorithm to find the hyperplane which maximizes the margin in N-dimensional space. Which is commonly used in classification tasks. The large data-set costs a computational expensive. To avoid this problem, we use stochastic gradient descent to optimize the SVM’s cost function. The linear kernel is chosen. The overall scores are low similar to the Naive Bayes algorithm. The SVM model receives an accuracy of 0.0854. It receives precision and recall of 0.0106 and 0.5636, respectively. Finally F1 score of 0.0209.

According to Pethe, Kim & Skiena (2020), the BERT model has successfully competed with all other baseline models, such as the C99 algorithm (Choi, 2000), the three-layer baseline perceptron model with 300 neurons in each layer (Badjatiya et al., 2018) and trained word2vec embeddings (Mikolov et al., 2013), and the neural model described by Badjatiya et al. (2018) that uses LSTM. Therefore, we used the BERT Break Point Prediction model in this article as the baseline model for comparative analysis. Pethe, Kim & Skiena (2020)’s model used a pre-trained BERT with dynamic programming algorithm that was fine-tuned with the dataset. This article’s method trained the BERT Break Point Prediction model as stated and illustrated in Pethe, Kim & Skiena (2020), albeit with an additional method and an additional step of splitting the training validation and test dataset. The BERT Break Point Prediction model received an accuracy score of 0.7719, which was lower than that of our proposed models. The BERT Break Point Prediction model also received lower precision, recall, and F1 scores of 0.6661, 0.6618, and 0.6640, respectively.

### Case study

This section will show a case study of the purposed ensemble paragraph level attention model. This ensemble paragraph level attention model outperforms other baselines since the model not only correctly predicted the chapter breakpoint which contains the chapter keywords, but also the model can predict the books without those chapter keywords. The Table 2 shows the examples in results of true-positive prediction, false-negative prediction, false-positive prediction, and true-negative predictions. Paragraph 1 and paragraph 2 in the Table 2 refer to two consecutive paragraphs. The examples are taken from the book called, “The Adventures of A Brownie,” written by Miss Mulock.

**Table 2:** A result of the ensemble paragraph level attention model by given two consecutive paragraphs from “The Adventures of A Brownie,” by Miss Mulock.

Paragraph 1	Paragraph 2	Result
… and brownie played no more tricks with any body–til the next time.	adventure the second brownie and the cherry-tree the “Next time” was …	tp
… keep i must until it crumble into dust. I took the wren’s nest: god forgive me!	a child’s smile a child’s smile–nothing more; quiet and soft and grave, and …	fn
… get all my folding done by bedtime, and have a clear day for ironing tomorrow.	but when she did fetch them in, having bundled them all together in the dusk …	fp
… thief might have got in, and wandered all over the house without being found out.	“Hurrah, here’s luck!” cried brownie, lossing his cap up in the air, and bounding …	tn

**Figure 3:**
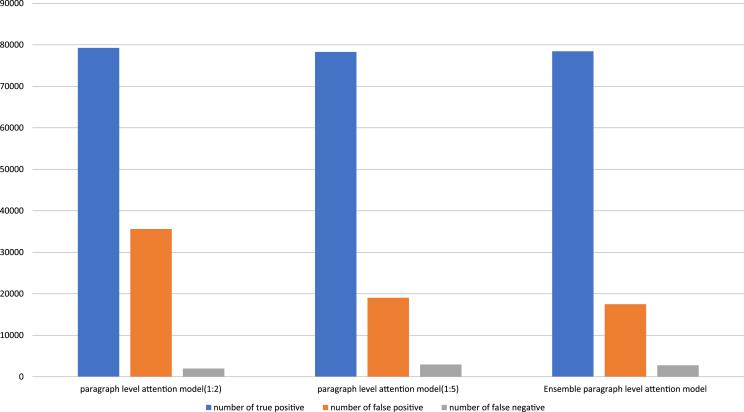
Number of true positive, false positive, and false negative predictions on each purposed model.

Figure 3 shows the number of prediction results on the paragraph level attention models and the ensemble paragraph level attention model. The ensemble paragraph level attention model can significantly decrease the number of false positive predictions, while changing an inconsiderable true positive and false negative predictions. As a result, the precision, which is inversely proportional to a number of false positive predictions, greatly increase, While the recall, which is inversely proportional to a number of false negative predictions, decrease insignificantly. Therefore, the F1 score of the ensemble paragraph level attention model outperforms the paragraph level attention models.

### Ablation study

**Figure 4:**
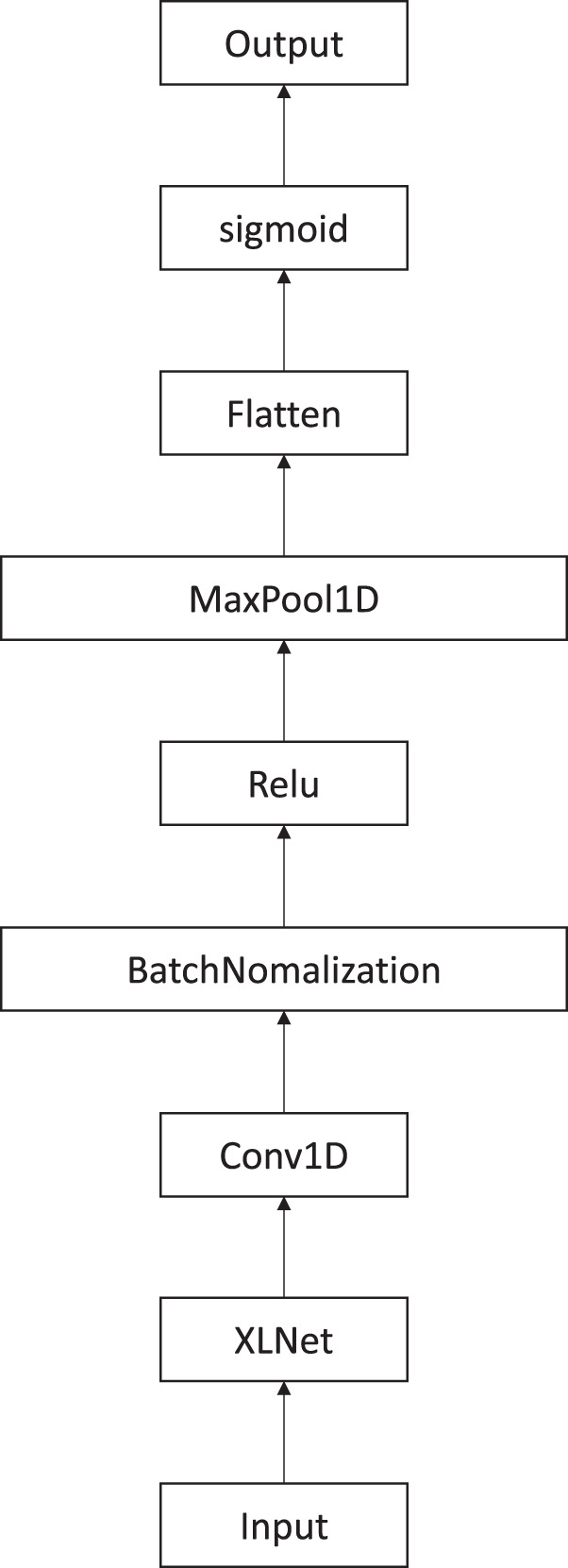
XLNet followed by CNN model to extract features.

In this section, we will prove that the proposed paragraph-level attention model indeed produced improved results. The proposed methods will be compared with the model (Fig. 4). This model did not include paragraph-level attention. It was created using XLNet and CNN. Instead of using two consecutive paragraphs separately as input, they were put together into the model. The model concatenated the two paragraphs together to create the input. This model connected the XLNet, CNN, batch normalization, rectifier activation function, and max pooling together. Then, the result matrices were flattened and we used the sigmoid activation function to get the binary classification. Although this model does not have a paragraph-level attention, it is created in the same fashion.

The results showed that the paragraph-level attention model greatly improved in overall results for the 1:2 ratio of positive to negative labels, and slightly increased for the 1:5 ratio. For the 1:2 ratio of positive to negative labels, the XLNet with CNN model had an accuracy score of 0.9849, which was 1% lower than the paragraph-level attention model’s score. The precision score of the XLNet with CNN model was 0.5344. The precision score of the paragraphlevel attention model was 0.6900, which showed an increase of 15%. The XLNet with CNN model’s recall score was 0.9855, which was only 1% higher than that of the paragraph-level attention model, which had a recall score of 0.9759. Ultimately, the F1 score of the XLNet with CNN model was 0.6930. The paragraph level attention model’s F1 score was 0.8084, which showed an improvement of 11%. This shows that the paragraph-level model’s overall score significantly improved.

Furthermore, we compared the models with positive and negative labels at the 1:5 ratio. The XLNet with CNN model received an accuracy score of 0.9948, which was lower than the paragraph-level attention model’s accuracy score of 0.9953. The precision score of XLNet with CNN was 0.7863, but the precision score of the paragraph-level attention model was 0.8043, which shows an improvement of about 2%. Next, the XLNet with CNN model’s recall score was 0.9574, and the paragraph-level attention model’s recall was 0.9637, which was only lower by 0.5%. The F1 score of the XLNet with CNN model was 0.8635, and the F1 score of the paragraph-level attention model was 0.8768, which showed an improvement of 1%.

The results show that the paragraph-level attention model had a major effect on increasing the precision score, but a slightly less significant effect on lowering the recall score. As a consequence, the F1 score significantly improved. Table 3 shows the results of the proposed model compared to the XLNet with CNN model.

**Table 3:** Evaluation of the proposed model compared to the XLNet with CNN model.

Model	Accuracy	Precision	Recall	F1
XLNet with CNN model (1:2)	0.9849	0.5344	0.9855	0.6930
Paragraph level attention model (1:2)	0.9920	0.6900	0.9759	0.8084
XLNet with CNN model (1:5)	0.9948	0.7863	0.9574	0.8635
Paragraph level attention model (1:5)	0.9953	0.8043	0.9637	0.8768

**Note:**

The paragraph-level attention model (1:2) is for the ratio of one to two training data labels. The paragraph-level attention model (1:5) is for the ratio of one to five training data labels. The XLNet with CNN model (1:2) is for the ratio of one to two training data labels. The XLNet with CNN model (1:5) is for the ratio of one to five training data labels.

## Conclusion

Each book contains unique structures and boundaries that a person can easily identify, including the book title, authors, table of contents, chapters, and footer. Writers give unique styles to their books whether they are novels, encyclopedias, journals, or textbooks. These factors all contribute to the fact that books as a collective form do not share the same type of structure. Even when using a rule-based algorithm, computers cannot perfectly segment the boundaries of each book chapter. Therefore, a learning algorithm is required to improve the accuracy of chapter prediction.

This article proposes a novel chapter segmentation method that uses paragraph-level attention. Our proposed method utilizes XLNet and CNN for feature learning, as well as a simple but effective attention mechanism to aggregate paragraph-level context information. Our thorough comparative analysis demonstrates that our method can achieve a much higher performance when compared with previous SOTA methods. More importantly, our ablation study validated the effectiveness of the proposed paragraph-level attention module, which may indicate that hierarchical semantic representation works better than using only token-level semantic features.

The proposed method shows specific improvements in accuracy, precision, recall, and F1 scores. The paragraph-level attention model increased the F1 score by 15% compared to the baseline of 0.8084. The ensemble paragraph level attention model showed a significantly improved F1 score of 0.8856, which was a 20% and 8% improvement from the baseline and the paragraph level attention model, respectively.

In addition to the results of our proposed method, we also suggest that machine learning models can still be improved to solve problems such as segmentation on the deeper multiple layers of seemingly simple, yet ambiguous, structures, such as book volumes, parts, chapters, and sub-chapters.

## Supplemental Information

10.7717/peerj-cs.1003/supp-1 Supplemental Information 1 The code files for the paragraph attention and ensemble model.

## Additional Information and Declarations

### Data Availability

The following information was supplied regarding data availability: The code is available in the Supplemental Files. The dataset is available at figshare: Virameteekul, Paveen (2022): dataset.zip. figshare. Dataset. https://doi.org/10.6084/m9.figshare.19361390.v1.
